# Comparison of Delay in Tuberculosis Diagnosis Between Migrants and Local Residents in an Eastern County of China: An Analysis of the Electronic Data Between 2015 and 2019

**DOI:** 10.3389/fpubh.2021.758335

**Published:** 2021-11-18

**Authors:** Wenhui Xiao, Bin Chen, Dajiang Huang, Olivia Chan, Xiaolin Wei, Lin Zhou, Guanyang Zou

**Affiliations:** ^1^School of Public Health and Management, Guangzhou University of Chinese Medicine, Guangzhou, China; ^2^Zhejiang Provincial Center for Disease Control and Prevention, Hangzhou, China; ^3^Center for Disease Control and Prevention, Cangnan County, Wenzhou, China; ^4^School of Public Health, University of Hong Kong, Hong Kong SAR, China; ^5^Division of Clinical Epidemiology & Institute of Health Policy, Management and Evaluation, Dalla Lana School of Public Health, University of Toronto, Toronto, ON, Canada

**Keywords:** migrants, tuberculosis, total diagnostic delay, patient delay, health system delay

## Abstract

**Introduction:** China continues to rank among one of the countries with the highest number of tuberculosis (TB) cases globally. Migrants are a particularly at-risk subgroup for TB and pose a challenge for case management in contemporary China. The early diagnosis and treatment of patients with TB are pivotal for effective TB control. This study investigates the delay in the TB diagnosis of migrants as compared with residents, to provide an evidence base for improved case detection and the better management of migrant patients with TB.

**Materials and Methods:** The data was collected from the Tuberculosis Information Management System (TBIMS) (2015–2019) in an eastern county of China. The total diagnostic delay, consisting of patient delay and health system delay, is defined as the interval between the onset of TB symptoms and the confirmation of TB diagnosis in the designated TB hospital. The comparison of the delay in the TB diagnosis between migrants and residents was conducted using a Mann-Whitney *U*-test and chi-square test. The difference in the delay curves between these two groups was examined using a log-rank test.

**Results:** Of 2,487 patients with TB, 539 (22%) were migrants. The migrants tended to be younger, presented with less severe conditions, received an initial diagnosis at prefectural and above-level hospitals. Compared with the local patients with TB, the migrant patients with TB had a longer median total diagnostic delay (30 vs. 9, *P* = 0.000) and a higher proportion of patients with this delay >28 days (52 vs. 13%, *P* = 0.000). Similarly, the migrant patients with TB also had a longer median patient delay (13 vs. 9, *P* = 0.000) and a higher proportion of patients with this delay >14 days (47 vs. 30%, *P* = 0.000), longer median health system delay (9 vs. 0, *P* = 0.000), and a higher proportion of patients with this delay >14 days (42 vs. 0.5%, *P* = 0.000) than the local patients with TB. The survival curves of delay showed that the longer the time interval was, the more likely the migrant patients with TB were to be diagnosed (*P* < 0.05).

**Conclusions:** Diagnosis is significantly delayed among migrant patients with TB. Our study highlights the importance of early screening and diagnosis for TB especially among migrants, to improve access and ensure better management for all patients with TB.

## Introduction

Tuberculosis is a communicable disease, the leading cause of death ranking above HIV/AIDS. Globally, an estimate of 10 million people fell ill with tuberculosis (TB), and 1.4 million people died from this disease in 2019 (1). It is generally believed that TB is a disease of poverty, as 98% of TB cases were reported from 121 low-and middle-income countries in the same year (1). China is listed as one of the 30 TB high burden countries in the world. In 2019, an estimated 8,33,000 people fell ill with TB in China, accounting for 8.4% of the global TB cases burden (1). Over the past two decades, there has been significant progress in TB control in China, with the incidence being halved and mortality reduced by more than 70% (1). Nevertheless, China is still facing tremendous challenges in TB control, including a high TB burden and difficulties in case detection and management among migrants.

In China, the migrant populations are mostly internal migrants (henceforth called migrants). In 2019, the number of migrants in China reached 236 million, accounting for one-sixth of the whole population (2). Most migrants in China have traveled from rural areas to prosperous economic regions to earn higher incomes and find better opportunities (3, 4). The large population of migrants and domestic flows have had considerable impacts on the TB epidemic in China. A national study reported that migrants accounted for one-fifth of all the TB cases nationwide between 2014 and 2015, representing 0.3 million migrant patients with TB (5). Migrants are always considered as an important vulnerable subgroup among patients with TB (6). Compared with residents, migrants face substantial problems in accessing medical care, insurance, and social security (7). Many studies reported poor health-service-seeking behavior among migrants in China (8–11), and these studies indicated the inequities of accessing health services among migrants and residents.

The early detection and timely treatment of migrant patients with TB are essential for effective TB control and are emphasized in the “End TB strategy” of the WHO (6, 12). Delays occurring during the process of diagnosis and treatment increase the probability of TB transmission and eventually result in a higher disease burden (13). In most places in China, patients with TB receive standardized diagnosis and treatment in the designated general hospital of the county, and other health facilities refer patients with TB or presumptive patients with TB to the designated TB hospitals for standardized diagnosis and treatment (14). Numerous studies have focused on the delay in TB diagnosis for migrants in China (13, 15–20). However, most of these studies in China were conducted in big cities such as Shanghai and Shenzhen. Few have studied the delay in TB diagnosis for migrants living in the counties. In a cross-sectional study conducted in 12 counties of Shandong Province, Zhou et al. reported a median total diagnostic delay, patient delay, and health system delay of 21, 10, and 8 days among migrant patients with TB (13). They also found that a proportion of 41 and 45% migrant patients with TB have patient delay and health system delay of >14 days (13). Also, current evidence regarding the probability of being diagnosed at different time intervals for patients with TB (including migrants and residents) is limited. Chen et al. assessed the probability of being diagnosed at different months since the onset of TB symptoms for patients with TB to understand the delay of diagnosis using survival analysis (21). This study analyzes the TB diagnosis delay among the migrant patients with TB as compared with local patients with TB registered between 2015 and 2019 in Cangnan County, Zhejiang province, to provide an evidence base for the improved case detection and better management of migrant patients with TB.

## Materials and Methods

### Study Design

This is a retrospective study of the diagnostic delay of migrant patients with TB as compared with local patients with TB over 5 years (2015–2019). The data was collected from the patient records exported from the Tuberculosis Information Management System of China (TBIMS) (TB special reporting system version 2.0).

### Study Setting

This study was conducted in Cangnan County of Wenzhou City, Zhejiang Province. Zhejiang is one of the most developed provinces in China and a key destination for migrants. Wenzhou is a city under the jurisdiction of Zhejiang Province. In 2019, Wenzhou had a total gross domestic product (GDP) exceeding the US $100 billion, ranking third out of 11 cities in Zhejiang province, with an average per capita GDP of $10,000 (22). Wenzhou City consists of four districts, five counties, and three county-level cities with a population of 9.3 million (22). Cangnan is a populous and well-off county in Wenzhou, with a population of 1.24 million and an average per capita GDP of $9,200 (22). In each county of Wenzhou City, there is a designated general hospital responsible for TB diagnosis and treatment, and in Cangnan, it is the Cangnan County People's Hospital.

### Data Collection

This study used the routine electronic practice data that were collected and recorded by TB health providers during TB consultation at the time of TB registration. This study selected the data of patients with TB registered from January 1, 2015, to December 31, 2019. The data was exported from TBIMS to Microsoft Excel (Microsoft, Redmond, Washington, United States) by the staff in the Center for Disease Control and Prevention (CDC) of Zhejiang Province and Cangnan. The data covers the demographics of patients with TB such as age, sex, occupation, patient source, level of the hospital for initial TB diagnosis, and household registration (migrant or local resident); clinical characteristics such as smear sputum results, cavity, TB severity (e.g., with large cavities or lesions in more than two lung lobes), treatment category (new or retreated cases), treatment outcomes; and health service-related information such as date of onset of TB symptoms, date of first health-care visit, and date of confirmed TB diagnosis.

### Definitions

In this study, patients with TB who did not have household registration in Cangnan County or who lived in Cangnan County for <6 months at the time of registration was classified as “migrant” in the TBIMS. In this study, we analyzed the total diagnostic delay, patient delay, and health system delay of migrant patients with TB as compared with local patients with TB. The total diagnostic delay (henceforth called total delay) is the sum of the patient delay and health system delay. The patient delay and health system delay is defined as the interval between the onset of TB symptoms and the first visit to a health facility, and the interval between the first visit to a health facility and confirmed TB diagnosis in the TB designated hospital, respectively (23). We used 28 days as a cut-off point for the analysis of total delay and 14 days as a cut-off point for the analysis of patient delay and health system delay based on previous studies (24–26). Tuberculosis diagnosis is mainly based on sputum smear examination, supplemented by sputum culture and X-Ray. To diagnose TB, suspected patients with TB need to have three samples of smear sputum to be collected and tested, i.e., a sample of “instant sputum” on the same day of TB clinic visit, a second sample of “night sputum” and a third sample of “morning sputum” next day (27).

### Data Analysis

The data were analyzed using SPSS 21.0 (SPSS, Inc., Chicago, United States). The demographics, clinical symptoms, and delay in TB diagnosis for the migrant and local patients with TB were depicted using descriptive statistics. The continuous variables were presented by the median and interquartile range (IQR), while the categorical variables were presented by counts and proportions. The delay in the TB diagnosis (including delay times and delay proportion) between the migrant and local patients with TB was compared using a Mann-Whitney U test and Chi-square test. In addition, we estimated the probability of the migrant patients with TB being diagnosed at different time intervals (e.g., 1^*^14 days) based on three diagnostic delay categories, as compared with the local residents, further testifying and comparing the diagnostic delay among these two cohorts. Hence, Kaplan-Meier survival curves were drawn to record the median times of total delay, patient delay, and health system across 5 years. The difference in these delay curves between migrant and local patients with TB was examined using a Log-rank test. A *p*-value of <0.05 was considered statistically significant.

## Results

### Demographics and Clinic Symptoms of Migrant TB Patients as Compared With Local TB Patients

A total of 2,487 TB cases were reported between 2015 and 2019 in Cangnan County, Zhejiang Province, including 539 migrants (22%) and 1,948 residents (78%). The migrant patients with TB were significantly younger than the local patients with TB (39 vs. 49 years, *p* < 0.05) and had a lower proportion of patients who were farmers (39 vs. 57%, *p* < 0.05), with a cavity (33 vs. 39%, *p* < 0.05), and severe cases (27 vs. 38%, *p* < 0.05). Compared with the local patients with TB, the migrant patients with TB had a lower proportion of patients who were referred to TB designated hospitals from other health facilities (35 vs. 77%, *p* < 0.05), and firstly diagnosed at county-level TB designated hospitals (35 vs. 99%, *p* < 0.05) over 5 years. In terms of treatment outcomes, the migrant patients with TB were more likely to end up with unfavorable outcomes than the local patients with TB (15 vs. 4%, *p* < 0.05). For instance, the proportion of the migrant patients who were unable to follow-up during the treatment was 7%, as compared with 0.6% of the local patients (Table 1).

**Table 1:** Demographics, clinical characteristics of migrant and local patients with TB from 2015 to 2019.

	**Migrant TB patients** **(***n*** = 539)**						**Local TB patients** **(***n*** = 1948)**						
	**2015**	**2016**	**2017**	**2018**	**2019**	**Total**	**2015**	**2016**	**2017**	**2018**	**2019**	**Total**	***p*** **-value**
Age (Median, IQR)	29 (22–49)	36 (25–52)	44 (31–56)	42 (26–54)	41 (28–54)	39 (26–53)	49 33–61)	46 (30–60)	51 (37–65)	49 (31–63)	52 (35–65)	49 (33–63)	0.000
Age >45	26 (29)	40 (40)	4 5 (44)	47 (40)	53 (41)	211 (39)	265 (55)	206 (51)	246 (64)	203 (56)	194 (61)	1,114 (57)	0.000
Male	61 (69)	71 (70)	69 (68)	85 (73)	94 (72)	380 (71)	349 (73)	295 (73)	287 (74)	259 (72)	242 (76)	1,432 (74)	0.164
Farmer	43 (48)	48 (48)	34 (33)	27 (23)	38 (30)	190 (35)	432 (90)	340 (85)	309 (80)	207 (57)	217 (69)	1,505 (77)	0.000
Smear positive	40 (45)	32 (32)	41 (40)	35 (30)	35 (27)	183 (34)	174 (36)	113 (28)	136 (35)	146 (40)	114 (36)	683 (35)	0.632
Cavity	39 (44)	37 (37)	37 (36)	31 (27)	34 (27)	178 (33)	182 (38)	153 (38)	156 (40)	136 (38)	134 (42)	761 (39)	0.012
Severe cases	38 (43)	33 (33)	33 (32)	17 (15)	22 (17)	143 (27)	172 (36)	150 (37)	155 (40)	127 (35)	130 (41)	734 (38)	0.000
Patient source^a^													0.000
Symptomatic visits	44 (49)	77 (76)	73 (72)	80 (70)	39 (30)	313 (68)	111 (23)	97 (24)	60 (16)	32 (9)	25 (8)	325 (17)	
Referral	41 (46)	23 (23)	24 (24)	22 (19)	34 (26)	144 (32)	368 (77)	305 (76)	323 (84)	329 (91)	291 (92)	1,616 (83)	
Treatment category													0.945
New	76 (85)	80 (79)	90 (88)	108 (92)	118 (91)	472 (88)	425 (89)	353 (88)	332 (86)	311 (86)	287 (91)	1,708 (88)	
Retreated	13 (15)	21 (21)	12 (12)	9 (8)	12 (9)	67 (12)	55 (11)	49 (12)	55 (14)	51 (14)	30 (9)	240 (12)	
Level of hospital for initial TB diagnosis													
County	48 (54)	36 (36)	36 (35)	29 (25)	38 (29)	187 (35)	479 (99)	400 (99)	386 (99)	361 (99)	315 (99)	1,941 (99)	0.000
Prefectural and above	41 (46)	65 (64)	66 (65)	88 (75)	92 (71)	352 (65)	1 (1)	2 (1)	1 (1)	1 (1)	2 (1)	7 (1)	
Treatment outcomes^b^													0.000
Treatment success	80 (90)	76 (75)	88 (86)	99 (90)	7 (70)	350 (85)	470 (98)	395 (98)	367 (95)	331 (95)	13 (62)	1,576 (96)	
Cured	36 (40)	20 (20)	37 (36)	29 (26)	1 (10)	123 (30)	167 (35)	110 (27)	122 (32)	132 (38)	1 (5.0)	532 (32)	
Treatment completed	44 (49)	56 (55)	51 (50)	70 (64)	6 (60)	227 (55)	303 (63)	285 (71)	245 (63)	199 (57)	12 (57)	1,044 (64)	
Unfavorable outcomes	9 (10)	25 (25)	14 (14)	11 (10)	3 (30)	62 (15)	10 (2.0)	7 (2.0)	20 (5.0)	17 (5.0)	8 (38)	62 (4.0)	
Lost to follow-up	5 (5.6)	12 (12)	8 (7.8)	4 (3.6)	0 (0.0)	29 (7.0)	0 (0.0)	0 (0.0)	7 (1.8)	3 (0.9)	0 (0.0)	10 (0.6)	
Treatment failed	1 (1.1)	2 (2.0)	1 (1.0)	0 (0.0)	0 (0.0)	4 (1.0)	6 (1.3)	1 (0.2)	6 (1.6)	4 (1.1)	3 (14)	20 (1.2)	
Diagnostic change	0 (0.0)	5 (5.0)	3 (2.9)	4 (3.6)	1 (10)	13 (3.2)	2 (0.4)	3 (0.7)	2 (0.5)	7 (2.0)	2 (9.5)	16 (1.0)	
Transfer to MDR	0 (0.0)	2 (2.0)	1 (1.0)	2 (1.8)	1 (10)	6 (1.5)	1 (0.2)	1 (0.2)	0 (0.0)	2 (0.6)	1 (5.0)	5 (0.3)	
Adverse reactions	0 (0.0)	0 (0.0)	0 (0.0)	1 (0.9)	0 (0.0)	1 (0.2)	0 (0.0)	0 (0.0)	0 (0.0)	0 (0.0)	0 (0.0)	0 (0.0)	
Died	1 (1.1)	1 (1.0)	0 (0.0)	0 (0.0)	1 (10)	3 (0.7)	1 (0.2)	2 (0.5)	4 (1.0)	0 (0.0)	0 (0.0)	7 (0.4)	
Other	2 (2.2)	3 (3.0)	1 (1.0)	0 (0.0)	0 (0.0)	6 (1.5)	0 (0.0)	0 (0.0)	1 (0.3)	1 (0.3)	2 (9.5)	4 (0.2)	

*Unless indicated otherwise, data are given as n (%)*.

a *In addition to symptomatic visits and referral, there were also 43% migrant patients with TB that came from close contact tracing in 2019*.

b *In this variable, we presented treatment outcomes of 2050 patients who stopped treatment before the data was exported from TBIMS*.

### Total Delay, Patient Delay, and Health System Delay of Migrant TB Patients as Compared With Local TB Patients

The median (IQR) total delay for the migrant patients with TB was 30 (11–64) days, significantly longer than the 9 (4–17) days for the local patients with TB over 5 years (*P* < 0.05). In addition, compared with the local patients with TB, the migrant patients with TB experienced significantly longer total delays every year from 2015 to 2019 (*P* < 0.05). The proportion with a total delay of >28 days for the migrant patients with TB was 52%, significantly higher than the 13% for the local patients with TB over 5 years (*P* < 0.05). In addition, compared with the local patients with TB, the migrant patients with TB had a significantly higher proportion of patients whose total delay was >28 days every year from 2015 to 2019 (*P* < 0.05) (Table 2).

**Table 2:** The total delay of migrant and local patients with TB from 2015 to 2019.

**Year**	**Days (Median, IQR)**			**Proportion (** ***n*** **, %)**		
	**Migrants**	**Local**	***p*** **-value**	**Migrants**	**Local**	***p*** **-value**
2015	29 (10–52)	9 (4–19)	0.000	45 (51)	63 (13)	0.000
2016	30 (12–60)	8 (4–16)	0.000	53 (53)	47 (12)	0.000
2017	38 (16–78)	10 (4–21)	0.000	61 (60)	63 (16)	0.000
2018	49 (21–105)	8 (3–18)	0.000	83 (71)	53 (15)	0.000
2019	17 (7–33)	9 (3–14)	0.000	38 (29)	31 (10)	0.000
2015–2019	30 (11–64)	9 (4–17)	0.000	280 (52)	257 (13)	0.000

The median (IQR) patient delay for the migrant patients with TB was 13 (4–34) days, significantly longer than the 9 (4–17) days for local patients with TB over 5 years (*P* < 0.05). In addition, compared with the local patients with TB, the migrant patients with TB experienced significantly longer patient delays every year from 2015 to 2018 *(P* < 0.05). However, the migrant patients with TB experienced shorter patient delays as compared with the local patients with TB in 2019 (4 vs. 9 days, *p* < 0.05). The proportion with a patient delay of > 14 days for the migrant patients with TB was 47%, significantly higher than the 30% for the local patients with TB over 5 years (*P* < 0.05). In addition, compared with the local patients with TB, the migrant patients with TB had a significantly higher proportion of patients whose patient delay was >14 days every year from 2015 to 2018 (*P* < 0.05). However, no significant difference was observed among these two groups regarding patient delay >14 days in 2019 (*P* > 0.05) (Table 3).

**Table 3:** Patient delay of migrant and local patients with TB from 2015 to 2019.

**Year**	**Days (Median, IQR)**			**Proportion** **(***n***, %)**		
	**Migrants**	**Local**	**P value**	**Migrants**	**Local**	***p*** **-value**
2015	13(6–31)	9 (4–19)	0.001	41 (46)	156 (33)	0.013
2016	17 (6–35)	8 (4–16)	0.000	54 (53)	112 (28)	0.000
2017	20 (6–53)	10 (4–21)	0.000	55 (54)	143 (37)	0.002
2018	28 (8–59)	8 (3–17)	0.000	68 (58)	106 (29)	0.000
2019	4 (0–15)	9 (3–14)	0.000	33 (25)	70 (22)	0.451
2015–2019	13(4–34)	9 (4–17)	0.000	251 (47)	587 (30)	0.000

The median (IQR) health system delay for the migrant patients with TB was 9 (0–25) days, which is significantly longer than the 0 (0–0) days for the local patients with TB over 5 years *(P* < 0.05). In addition, compared with the local patients with TB, the migrant patients with TB experienced significantly longer health system delays every year from 2015 to 2019 (*P* < 0.05). The proportion with a health system delay of >14 days for the migrant patients with TB was 42%, significantly higher than the 0.5% for the local patients with TB over 5 years (*P* < 0.05). In addition, compared with the local patients with TB, the migrant patients with TB had a significantly higher proportion of patients whose patient delay was >14 days every year from 2015 to 2019 too (*P* < 0.05) (Table 4).

**Table 4:** Health system delay of migrant and local patients with TB from 2015 to 2019.

**Year**	**Days (Median, IQR)**			**Proportion** **(***n***, %)**		
	**Migrants**	**Local**	***p*** **-value**	**Migrants**	**Local**	***p*** **-value**
2015	1 (0–21)	0 (0–0)	0.000	34 (38)	2 (0.4)	0.000
2016	11 (0–23)	0 (0–0)	0.000	46 (46)	2 (0.5)	0.000
2017	16 (0–29)	0 (0–0)	0.000	53 (52)	1 (0.3)	0.000
2018	15 (0–42)	0 (0–0)	0.000	60 (51)	3 (0.8)	0.000
2019	5 (0–15)	0 (0–0)	0.000	35 (27)	2 (0.6)	0.000
2015–2019	9 (0–25)	0 (0–0)	0.000	228 (42)	10 (0.5)	0.000

### Survival Curves

**Figure 1:**
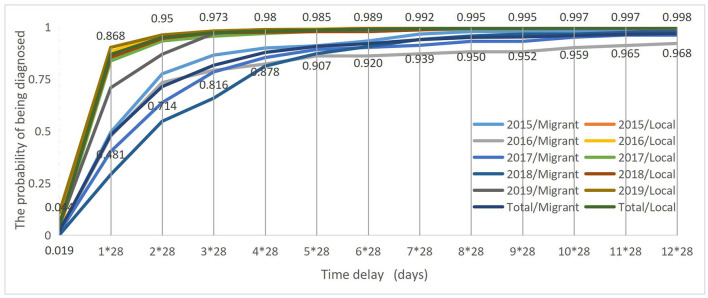
Total delay curves between migrant and local patients with TB from 2015 to 2019.

The log-rank test showed that the total delay curves significantly differed between the migrant and local patients with TB over 5 years (Log-rank test χ^2^ = 268.409, *p* < 0.001). The probability of the migrant patients with TB being diagnosed within 28 days since the onset of symptoms was 48% as compared with the 87% of local patients with TB. Furthermore, it took up to 8^*^28 days for 95% of the migrant patients with TB to be diagnosed since the onset of TB symptoms, and 2^*^28 days for 95% of the local patients with TB to be diagnosed since the onset of TB symptoms (Figure 1).

**Figure 2:**
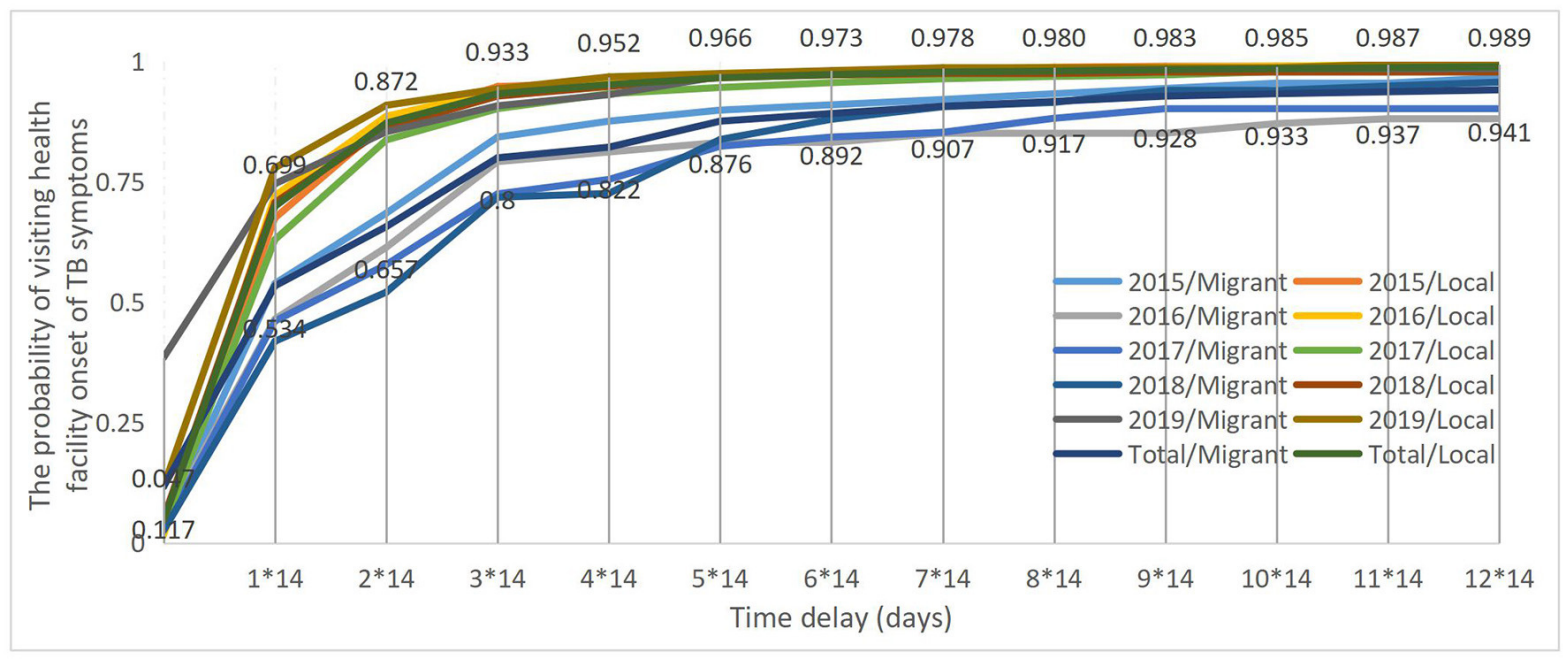
Patient delay curves between migrant and local patients with TB from 2015 to 2019.

The log-rank test showed that the patient delay curves significantly differed between the migrant and local patients with TB over 5 years (Log-rank test χ^2^ = 78.135, *p* < 0.001). The probability of the migrant patients with TB visiting health facilities within 14 days of onset TB symptoms was 53% as compared with the 70% of the local patients with TB. Furthermore, it took up to 12^*^14 days and longer for 95% of the migrant patients with TB to visit health facilities since the onset of TB symptoms, and 4^*^14 days for 95% of the local patients with TB to visit health facilities since the onset of TB symptoms (Figure 2).

**Figure 3:**
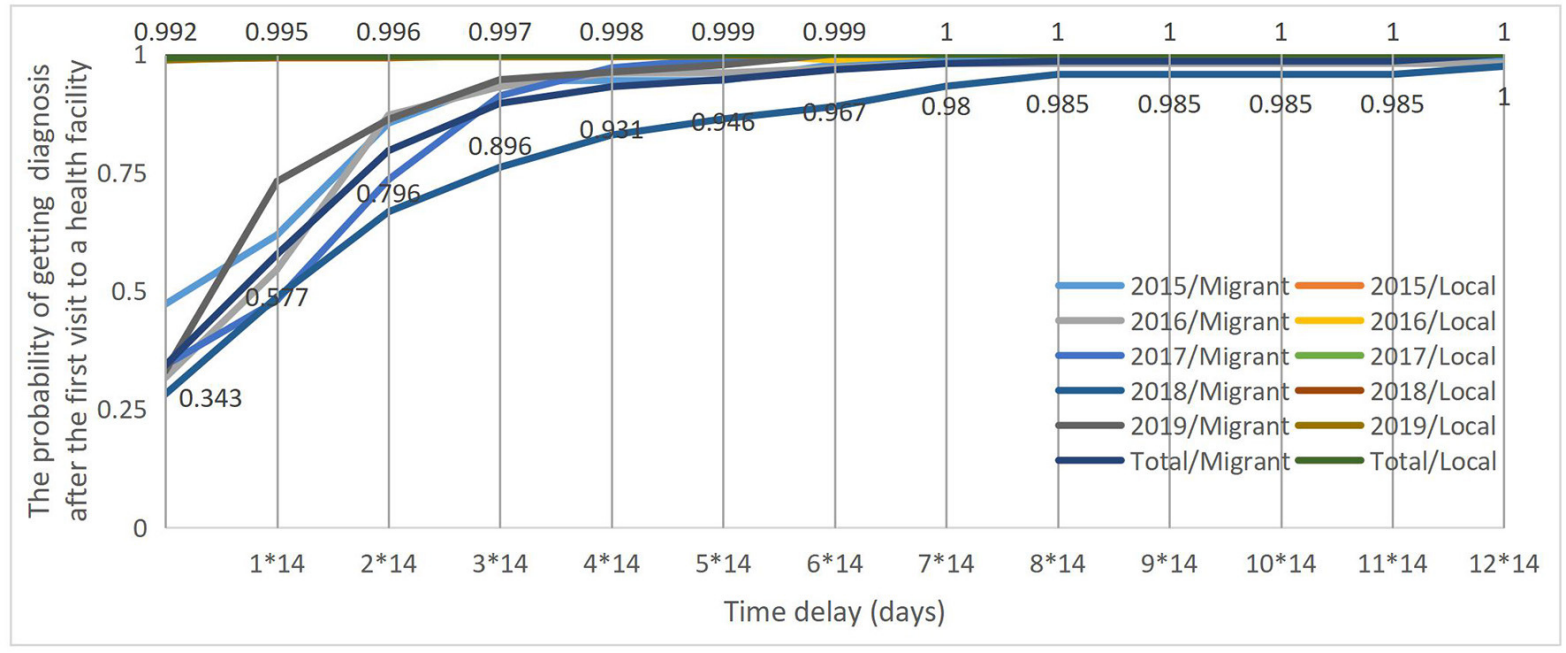
Health system delay curves between migrant and local patients with TB from 2015 to 2019.

The log-rank test showed that the health system delay curves significantly differed between the migrants and local patients with TB over 5 years (Log-rank test χ^2^ = 1,169.030, *p* < 0.001). The probability of the migrant patients with TB getting a confirmed diagnosis within 14 days after the first visit to a health facility was 58% as compared with the 99.5% of the local patients with TB. Furthermore, it took up to 5^*^14 days and longer for 95% of the migrant patients with TB to get a confirmed diagnosis after their first visit to a health facility. However, the health system delay curve for the local patients with TB was the infinitely approaching point “1” from the beginning, which showed that the local patients with TB have nearly 100% probability of getting a confirmed diagnosis on the same day when they first visited a health facility (Figure 3).

## Discussion

### Summary of Findings

In this study, we found that migrant patients with TB tended to be younger, non-farmer, have less severe conditions, receive an initial diagnosis at prefectural and above-level hospitals. The median total delay, patient delay, and health system delay for migrant patients with TB were 30, 13, and 9 days, respectively, as compared with 9, 9, and 0 days for local patients with TB. Compared with local patients with TB, migrant patients with TB had a higher proportion of patients with a total delay of >28 days, patient delay of >14 days, and health system delay of >14 days. The survival curves of delay showed that the longer the time interval was, the more likely the migrant patients with TB were to be diagnosed.

### Comparison With Literature

Migrants remain one of the most important vulnerable subgroups in the context of the TB epidemic. Tuberculosis control in migrants is pivotal for countries to progress toward TB elimination in accordance with the WHO END TB strategy (12). In this study, we found that migrants accounted for 22% of all the registered TB cases between 2015 and 2019 in a populous County of an Eastern Province in China. This was slightly higher than the 18% reported by Li et al. in a national study during 2014–2015 (5), yet was much lower than the 72% in the study of Zhao conducted in Shanghai, a metropolis experiencing rapid economic development where the majority of the population are migrants (19).

Similar to the previous studies (8, 28–30), the migrants in our studies were younger and presented with less severe clinical symptoms as compared with the residents. This phenomenon is associated with the “healthy migrant effect” and reflects the self-selection of younger and healthier individuals to pursue higher incomes and better opportunities in more economically developed areas. In China, the tendency for people to seek health care at higher-level hospitals is universal (31). Our findings substantiate the phenomenon as more migrants seek medical care in tertiary hospitals compared with residents. One possibility is the geographic proximity of migrants to the County Center, with easier access to tertiary hospitals in the city. Similar to previous studies (32), we found that migrant patients with TB had poorer treatment outcomes, especially with a higher lost-to-follow-up rate than local patients. This is mainly due to the “floating” characteristics of migrants and the lack of health insurance among the migrants in the host cities (33).

The main purpose of this study is to examine the delayed diagnosis of migrant patients with TB as compared with local patients with TB. In this study, we reported a median total delay of 30 days for the migrant patients with TB, which is between the 21 days reported by Zhou et al. in the counties of Shandong and 46 days reported by Xiao et al. in the counties of Zhejiang (13, 23). Our study found that migrant patients with TB had a longer total delay as compared with local patients with TB, which is similar to an earlier study focused on patients with TB with diabetes mellitus (DM) comorbidity (23). We also found that migrant patients with TB had a higher proportion of patients with a total delay of >28 days.

Our study reported a median patient delay of 13 days and a proportion of 47% with patient delay >14 days for migrant patients with TB. Previous studies have reported a median patient delay ranging from 10 to 21 days and a proportion ranging from 39 to 68% with patient delay >14 days for migrant patients with TB in China (13, 15, 16, 18, 19, 23, 34). The present study found that migrant patients with TB had a longer patient delay as compared with local patients with TB. However, two studies conducted in Shanghai reported no significant difference between migrant and local patients with TB regarding patient delay time (16, 19). One possible explanation for this discrepancy is that migrants in Shanghai had better healthcare access and resources than in other cities (16). Similar to the study by Long et al. (18), our study found that migrant patients with TB had a higher proportion of patients with patient delay >14 days. The barriers for migrant access to TB diagnosis included lack of awareness, lack of family and social support, lack of time, and inconvenience of medical insurance reimbursement (9–11, 18). It was noteworthy that our study reported shorter patient delays for migrant patients with TB as compared with local patients with TB in 2019. The significantly improved patient delay among migrant patients with TB is possibly due to the active case detection strategy implemented by the local CDC. In 2019, nearly half of the migrant patients with TB were detected from close contact tracing, while in other years they were mainly detected from symptomatic visits. Our study highlights the importance of promoting health education and improving health awareness, especially among migrants to reduce patient delay.

Our study reported a median health system delay of 9 days and a proportion of 42% with health system delay >14 days for migrant patients with TB. This finding is consistent with the results from previous studies, which reported a median health system delay ranging from 8 to 11 days and a proportion ranging from 27 to 45% with health system delay >14 days for migrant patients with TB in China (13, 15, 16, 19, 23). Similar to a previous study (23), we found that migrant patients with TB had longer health system delays and a higher proportion of patients with health system delays >14 days. One possible explanation for this is that migrant patients with TB tended to present light symptoms of TB which could be neglected by some health providers on their first visit to health facilities. Therefore, they need to make several visits to health facilities before being classified as having presumptive TB and referred to a TB designated hospital for TB diagnosis confirmation. Furthermore, some migrants might choose to seek health care back in their hometown when they are classified as presumptive TB. Our study indicates the need to strengthen TB diagnostic training and education for health providers, especially those working at prefectural and higher levels, when consulting migrants with suspected TB symptoms, to provide timely screening or referral to the TB designated hospitals.

Very few studies have employed survival analysis into the research of delay in TB diagnosis among patients with TB. The study of Chen et al. seemed to be the first study to analyze the diagnostic delay of patients with TB using survival analysis in China (21). In this study, we employed survival analysis to study the probability of being diagnosed at different time intervals for migrant patients with TB as compared with local patients with TB. Our study found that the longer the time interval, the more likely migrant patients with TB are to be diagnosed as compared with local patients with TB. This again confirms that more serious delays in diagnosis occurred in the migrant patients with TB than the local residents.

### Limitations

Our study has several limitations. First, this study was only conducted in only one county of China, hence the generalizability of our findings is limited. However, with a larger sample size including all registered TB cases from 2015 to 2019, our study provides solid data on the diagnostic delay for migrant patients with TB as compared with local patients with TB. Second, the time of onset of TB symptoms and first visit to a health facility was based on the self-reported information of patients with TB during clinical consultations, thus recall biases might exist. Third, the diagnosis of TB is sometimes difficult due to the confusion between TB and other diseases like lung cancer. In this case, the delay may be prolonged, in addition to the delay caused by the diagnostic process itself as it takes 2 days for three samples of smear sputum to be tested before TB confirmation. In other words, our analysis of health systems delay may dilute the diagnostic process when patients arrive at the TB clinic. Future studies should make differences between provider delay (from the first contact of the health facilities to the visit of the TB clinic) and confirmation delay (from the first visit of the TB clinic to the confirmation of TB). Finally, our study discloses the patterns of differences in diagnostic delays and time points between migrant and local patients for 5 years. Previous studies have suggested that factors such as socio-demographic and clinical characteristics could also influence the patient and health systems delay (21, 35–37). Further studies could be conducted to identify the impact of these confounding factors on the differences in the diagnostic delays between these two groups of patients.

## Conclusion

Diagnosis is significantly delayed among migrant patients with TB. Our study highlights the importance of early screening and diagnosis for TB especially among migrants, to improve access and ensure better management for all patients with TB.

## Data Availability Statement

The data analyzed in this study is subject to the following licenses/restrictions: Information in our database is confidential. Requests to access these datasets should be directed to Guanyang Zou, zgy1021@hotmail.com.

## Ethics Statement

The studies involving human participants were reviewed and approved by Zhejiang Provincial Center for Disease Control and Prevention. Written informed consent for participation was not provided by the participants' legal guardians/next of kin because: This is a patient record review study with data exported from China's Tuberculosis Information Management System. Data recorded in the system and used for this study are collected during TB registration and consultation.

## Author Contributions

GZ, WX, and LZ conceived and designed the study. BC and DH participated in data collection and analysis. GZ and WX were major contributors in writing the manuscript. OC and XW provided constructive suggestions on the study. All authors contributed to the article and approved the submitted version.

## Funding

This study was supported by the National Social Science Foundation of China (Grant Number 20&ZD122) and the Zhejiang Provincial Science and Public Welfare Project (LGF19H260004). The funding source was not involved in the design of the study and the collection, analysis and interpretation of data, and writing the manuscript.

## Conflict of Interest

The authors declare that the research was conducted in the absence of any commercial or financial relationships that could be construed as a potential conflict of interest.

## Publisher's Note

All claims expressed in this article are solely those of the authors and do not necessarily represent those of their affiliated organizations, or those of the publisher, the editors and the reviewers. Any product that may be evaluated in this article, or claim that may be made by its manufacturer, is not guaranteed or endorsed by the publisher.

## Abbreviations

TB: Tuberculosis
WHO: World Health Organization
TBIMS: China's Tuberculosis information management system
GDP: Gross Domestic Products
CDCs: Center for Disease Prevention and Control
IQR: Interquartile Range.
